# Parents’ experiences of living with, and caring for children, adolescents and young adults with Mucopolysaccharidosis (MPS)

**DOI:** 10.1186/s13023-016-0521-0

**Published:** 2016-10-10

**Authors:** S. Somanadhan, P. J. Larkin

**Affiliations:** 1 Temple Street Children’s University Hospital, Dublin, Ireland; 2 University College Dublin (UCD) School of Nursing, Midwifery and Health Systems, Belfield, Dublin, Ireland; 3 Clinical Nursing (Palliative Care), Children’s Nursing, All-Ireland Institute of Hospice and Palliative Care, UCD School of Nursing, Midwifery and Health Systems and Our Lady’s Hospice & Care Services , Belfield, Dublin, Ireland

**Keywords:** Mucopolysaccharidosis, Parent, Children, Experience, Adolescent, Young Adult, Qualitative research, Phenomenology, Hurler syndrome, Hunter syndrome, Sanfilipo syndrome, Maroteaux-Lamy syndrome, Rare Disease

## Abstract

**Background:**

Many rare diseases of childhood are life-threatening and chronically debilitating, so living with a rare disease is an on-going challenge for patients and their families. MPS is one of a range of rare inherited metabolic disorders (IMDs) that come under category 3 of life-limiting conditions, where there is no curative treatment available at present. Although the study of rare diseases is increasingly novel, and of clinical importance to the population, the lack of empirical data in the field to support policy and strategy development is a compelling argument for further research to be sought.

**Methods:**

This qualitative hermeneutic phenomenological study explored and interpreted Irish parents’ experiences of living with and caring for children, adolescents and young adults with MPS and the impact of these diseases on their day to day life. A purposively selected sample of parents’ attending the Irish National Centre for Inherited Metabolic Disorders was invited to participate in serial in-depth interviews.

**Results:**

A total of eight parents’ (*n* = 8) of children with a range of MPS disorders aged from 6 months to 22 years (MPS I Hurler syndrome, Scheie syndrome), MPS II (Hunter syndrome), MPS III (Sanfilipo syndrome) and MPS VI (Maroteaux-Lamy syndrome) were interviewed at three time points over a 17 month period. The main themes identified during data analysis were described as living with MPS, living with a genetic rare disease, the stigma of a rare condition, MPS as encompassing multiple diseases, Unknown future, hospital vs. home, experience of waiting, a tough road ahead, and things in their day-to-day life with MPS. They spoke of their child’s Quality of Life (QoL), their healthy children’s wellbeing, and for some, the impact on their own physical and psychological wellbeing. They also reflected on issues of stigmatisation and isolation in their experience of living with a child with a rare disorder.

**Conclusion:**

This study’s findings reflect the wider literature on the impact of rare diseases, which have also indicated how caring for someone with MPS, a condition that is chronic, progressive and degenerative can impact on all dimensions of the family’s life. Analysis of the findings using a hemenutic pheomenology perspecitve suggest that parents of children with MPS experience multiple cyclical movements across all five human lived existential experience, and they gradually develop ways to incorporate MPS in their day to day life. It was also evident that all the carers in this study experienced a range of uncertainties, with parents using terms such as ‘no man’s land’ and ‘future is unknown’ to describe their world.

## Background

Mucopolysaccharidoses (MPS) is one of the many rare inherited metabolic disorders (IMDs) which fall under category 3 of life-limiting conditions [1–4, 5]. It is caused by the body’s inability to produce specific lysosomal enzymes. This defective or missing lysosomal enzyme causes large amounts of complex sugar molecules or glycosaminoglycan’s (GAG’s) to accumulate in harmful amounts in the body's cells and tissues [1–4]. This result in progressive cellular damage, which in turn leads to an array of manifestations that worsen with age, and can affect multiple organ systems, leading to cognitive impairment and eventually resulting in severe morbidity and premature death [1–4]. It is important to understand that in almost all cases of MPS, the clinical feature(s) may not be visible at birth, and these children apparently have normal development in the first years of life but deteriorate as storage of GAGS affects organs and tissues [1–3, 6]. Except for MPS II, MPS are a genetic disorder inherited in an autosomal recessive pattern affecting both males and females [1–4]. Within MPS, the wide-ranging clinical spectrum and varying degree of severity mean that diagnosis can often be delayed [1, 3]. It has been reported that early diagnosis and treatment can improve outcomes in MPS patients [7, 8]. Transplantation of stem cells using bone marrow (Bone Marrow Transplant)(BMT), haematopoietic stem cell transplantation (HSCT) or umbilical cord blood (UCBT) has been shown to be effective in slowing disease progression in certain types of MPS [1–3, 9–11]. In other types of MPS (I, II and VI), enzyme replacement therapy (ERT) in the form of supportive treatments has shown benefits regarding GAG’s reduction and increased distance walked as part of the 6-minute walk test [1–3, 9–11]. Children with Mucopolysaccharidoses (MPS) and their families anticipate an unknown lifespan since it is a progressive condition with a lack of curative treatment options available at present, where treatment is exclusively palliative and may commonly extend over many years [5].

A comprehensive review of the literature was undertaken, focusing on the experience, impact, and consequences of caring for a child with MPS. Currently, the empirical body of knowledge on MPS is focused on the pathophysiological and genetic aspect of the disease rather than parents’ rare disease journey and their experience of living with a child with this condition. A small number of studies on the impact of parenting a child with MPS III [12–17] and MPS I [18] were identified and these studies report the experience as devastating, with a heavy burden being placed on parents due to the child’s severe behavioural and physical symptoms, as well as the progressive nature of the condition itself. However, no literature specifically examining the parental experience of caring for a child, adolescent and young adult across the spectrum of MPS was found. One Canadian study identified and explored the lived experience of parents with children diagnosed with MPS, who had received Enzyme Replacement Treatment (ERT), but were waiting for ongoing ERT funding to complete treatment [19]. Developments in advanced supportive treatment options such as ERT, BMT may have made dramatic changes in the quality of life (QoL) for individuals with certain forms of MPS. The WHO defines the quality of life as a *“broad ranging concept affected in a complex way by the person's physical health, psychological state, the level of independence, social relationships, personal beliefs and their relationship to salient features of their environment”* [20] (p. 1). Despite these developments, in most cases symptoms associated with the disease can affect multiple areas of daily living and bring major impairment to the individual’s lifespan [2, 4].

Recent studies recommend further research into the experience of caring for children with rare life-limiting conditions, investigating the needs of families for the long-term care of their child through adolescence to adulthood, and the specific services required [13, 14, 21]. The UK Strategy for Rare Diseases [22] and the National Rare Disease Plan for Ireland [23] and many more rare diseases national strategies [24, 25] across European member states highlight the importance of engaging and involving patients and their families in research and incorporating the patient and carers voices into the policies and services that affect them. These strategy documents have also highlighted the importance of establishing a variety of research approaches to rare diseases. Qualitative healthcare research, which can be tailored to explore the experience of living with a rare condition and the challenges patients and their caregivers face in their daily life, is becoming increasingly important. Given the lack of current evidence, there is a need to make explicit plan in developing and promoting best practice in care for the families of children with MPS, and in doing so make their lives more understandable to the wider healthcare audience.

The aims of this study were:

1. To understand and interpret parents’ experience of living and caring for a child with MPS.
2. To examine the knowledge and understanding of MPS from the perspective of parents.
3. To explore the impact of regular hospitalisation of children living with Mucopolysaccharidoses (MPS) on family life.

### Methods

During this study, some qualitatively-orientated research methodologies were examined in detail. Interpretive qualitative research often reflects questions about social aspects of health and illness of “what,” “how” or “why”? [26]. It is helpful where little is known about a topic or evidence is sparse. Qualitative research also explores the context of participants’ subjective experience. A hermeneutic phenomenological approach, informed by the philosophical constructs of Heidegger [27] Gadamer [28, 29] and Van Manen [30–32] was undertaken to study the complexities of a challenging life world [33]. Hermeneutic phenomenology is concerned with the study of lifeworld or human experience as we live it [30–33]. As such, this specific methodology enables a deeper understanding situated within parents’ day-to-day lives, managing and living with this condition in a family context, and the factors that both enhance and inhibit that experience [34]. Van Manen, [29, 30] proposed that *“hermeneutic phenomenological research is a search for the fullness of living”* (pp.12). In this case, it is a search for the way parents experiences the world as parents, and a search for what it is to be parents of a child, adolescent or young adult with MPS as a progressive life-limiting condition. Through a hermeneutic phenomenological approach, the uniqueness of this parent group was respected and validated, and the opportunity was given to share what may be concealed and unknown in their experience. In this study, utilising hermeneutic phenomenology enabled access to parents’ reflection in a particular moment of time on their lived experience and enabled a deeper understanding of the day-to-day challenges of living with a child, adolescent and young adult with an MPS disease.

### Recruitment

Given the rarity of the disease and relatively low numbers of children diagnosed each year, families were recruited from the outpatient clinic of the National Centre for Inherited Metabolic Disorders (NCIMD), Ireland where access to this specialist cohort group was more readily available.

**Inclusion Criteria**

- A mother or father of a child, adolescent or young adult (0–24 years old), with a diagnosed MPS condition (I-IX)
- Parents of children who attend the NCIMD
- Parents currently residing in the Republic of Ireland and receiving treatment for their child in Ireland
- Those able to communicate through English.

**Exclusion Criteria**

- Women who are pregnant at the time of data collection were excluded from the study. The recall of past experiences coupled with the fact that MPS is inherited in an autosomal recessive pattern, could potentially cause distress and exacerbate concern that the expected baby might be diagnosed with the same condition.

### Data collection and analysis

A total of eight parents’ (*n* = 8) participated in this study over a 17-month period. Data was collected through three time point serial interviews, a total of 19 in-depth, digitally-recorded interviews were completed, and each interview lasted for 60–120 min. Among these, one of the families only attended one set of interviews because of their children were subsequently admitted to hospital for treatment outside Ireland. All interviews were conducted in the hospital setting as a requirement of ethical approval for the study. At the time of interview, these parents had children aged between 6 months to 22 years, diagnosed with the following range of MPS disorders: MPS I syndromes (Hurler syndrome, Scheie syndrome), MPS II (Hunter syndrome), MPS III (Sanfilipo syndrome) and MPS VI (Maroteaux-Lamy syndrome). MPS IV (Morquio syndrome) was not included in the final sample. The small number of adults living with MPS IV was excluded from the study due to the age of this patient cohort (over 24 years). The parent of an adolescent with MPS IV initially agreed to participate in this study but withdrew at a later stage because of the long journey involved in attending for interview. All interviews were conducted in the hospital setting in order to meet the requirement of the research and ethics committee recommendation may have mitigated in accessing a complete sample, given that some families lived a considerable distance from the hospital. The serial interview approach provided an opportunity for the researcher and the parents to return to and reflect on the themes raised at the previous interview, thus enabling a process of interpretive insight [30–32]. The thematic analysis involved the use of Van Manen’s [31] three approaches: (1) the holistic or sententious approach, (2) the selective or highlighting approach, and (3) the detailed or line- by-line approach, were used. This process helped to identify and isolate key thematic aspects of the study phenomenon to prompt subsequent interviews.

MPS being a rare “orphan” disease, which affects a very small percentage of the population, the topic of anonymity and confidentiality has been a major concern among rare disease population-based research groups [35] Confidentiality was, therefore, a priority for these family units, and their identities were protected at all times, to prevent any breach of confidentiality. A code was therefore assigned to each family, and these codes were used throughout the data analysis. These codes are found at the end of each interview quote cited in the result section. For example, “F1” stands for “Family 1”, and the letters “A, B and C” stands for the “1st, 2nd, and 3rd interviews”, respectively.

Van Manen’s [31, 32] five existential themes emerged as a reflective data analysis framework following thematic analysis of interview transcripts. These five fundamental life world themes described the way humans experience the world, and these are *“Lived relation, Lived body, Lived space, Lived time, and Lived things”* [31, 32] underpinned the data analysis and meaningful expression of the parents day to day experience of living with MPS. Table 1 below demonstrates Van Manen’s [31, 32] five Fundamental Existential of Human Experience.

**Table 1:** Van Manen’s [29, 30] Five Fundamental Existential of Human Experience

Existential Theme	Description
Lived Relation	*“Is the lived relation we maintain with others in the interpersonal space that we share with them”* (Van Manen, 2007, pp. 104). How we relate to one another within an interpersonal shared space.
Lived Body	Lived body *“refers to the phenomenological fact that we are always bodily in the world’*” (Van Manen, 2007, pp. 102). The experience of being a physically or bodily in the world.
Lived Space	Van Manen (2007, pp.102) refers to lived space (spatiality) as a *“felt space … “.*The experience of space in which we find ourselves affects the way we feel.
Lived Time	*“Lived time refers to subjective time as opposed to clock time or objective time … and lived time is also our temporal way of being in the world”* (Van Manen, 2007, pp. 104).
Lived Things	*“The things are our world in its material thing like reality” … How are “things” experienced and how do the experiences of things and world contribute to the essential meaning of the phenomenon”* (Van Manen, 2014, pp. 307).

## Results

**Fig. 1:**
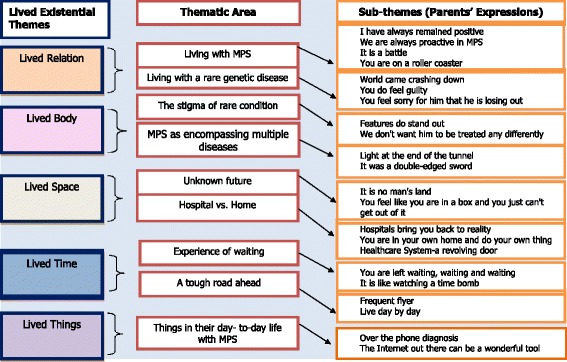
Summarises the themes and subthemes of the lived experience of parents of children, adolescents, young adults with MPS

Nine themes and 22 corresponding subthemes (parents’ expressions) were identified during data analysis. The main themes identified during data analysis were described as living with MPS, living with a genetic rare disease, the stigma of a rare condition, MPS as encompassing multiple diseases, Unknown future, hospital vs. home, experience of waiting, a tough road ahead, and things in their day-to-day life with MPS. The following sections will discuss these themes in the context of Van Manen’s five lived existential themes [31, 32] utilising selected interview extracts to illuminate that experience in the parental voice. Figure 1 below summarises the themes and subthemes of the lived experience of parents of children, adolescents, young adults with MPS.

### Lived relation

The existential theme of lived relation offered insight into the relational aspects of families’ day-to-day experience of living with MPS. The families in this study described the dynamics of relationships in terms of both family relations, reflecting their immediate family and close friends in a supportive care capacity, and of relations with the healthcare system and services, as a non-relational relationship. The two main themes of lived relation were: living with MPS and living with a rare genetic disease.

#### Living with MPS

The experience of living with MPS evoked strong emotions in these families, and they described life as characterised by times of uncertainty and ambiguity. They often described their emotional experience of living with MPS as being upset, confused, frustrated, and devastated. Some participants described their life as a roller coaster and living with MPS as a constant battle. Parents wanted to be proactive even though they knew that there was no cure for their child’s illness. They discussed occasions on which they had forced themselves to retain a positive outlook to keep the family together. One parent described her life as

*“Exceptionally busy, challenging, the frustrating challenges in coordinating appointments, coordinating doctors, and trying to fit everything in”* (FA01, pp. 11)

Four subthemes that defined this experience of living with MPS were identified from parents’ own expressions: “*I have always remained positive*”, “*we are always proactive in MPS*”, “*it is a battle*”, and “*you are on a roller coaster*”.

Unlike other types of MPS, MPS I (Hurler syndrome) patients have the potential supportive treatment of BMT, and that can have a positive impact on their day-to-day lived experience. The extract below outlines how the parent of a child with MPS I (Hurler syndrome), who had a BMT procedure, (F7) spoke about being positive for their child:

*“I mean there is not much we can do, we have to get on with life, and we are not going to give [our son] back no matter what now, that kind of way, he is such a great little boy, and we are so happy with him. We are positive for him, and we hope that we can help him do so much in his life, that kind of way so that is all we can do.” (FB07, pp.9) (F7 2* ^*nd*^ *Interview)*

Parents reports of focusing on the good, rather than bad aspects of being a parent of a child with MPS, and that thinking positively demonstrates the use of reframing strategies by parents whereby they replaced negative emotions with positive affirmations about their child and caregiving. Parents in this study have also reported that “*life is like a roller coaster*”, using this phrase to describe their experience of fluctuating between the good and bad and to reflect the progressive nature of their child’s illness.

*“…I don't know; it is how you cope and what state of mind, but emotionally you go through roller coasters, it is a roller coaster.” (FA01, pp. 14) (F1, 1* ^*st*^ *Interview)*

Families in this study reported that their marital relationships had become markedly strained. One of the families separated following their child’s diagnosis.

*“He is upset but [son's] dad has always been in denial, since the day he was born and we found out all this news he could never accept it, he was just always in denial. He said that he was fine because he started walking and he could feed himself and he ended up dressing himself, putting his trousers on himself, so his dad could never accept that he was a sick child, that he was terminal. He just can’t accept it. It upsets him but to him he is just like a normal child with a few learning disabilities. I don't think he will ever be able to accept what [son] has and what he is going through. That is his way of dealing with it” ((FA06, pp. 11) (F6, 2* ^*nd*^ *Interview)*

In this study, parents used the term roller coaster as a metaphor to describe the way their emotions changed over time. This term was used to describe the emotional impact of illness and the effect of the day to day experience of living with and caring for their child with MPS.

#### Living with rare genetic diseases

Parents described their experience of living with a rare genetic disease in terms of devastation, guilt, and concern regarding the ability of their child to engage fully with life. Families in this study reported that they were devastated by the enormity of the debilitating nature of their child’s illness and the impact on their child’s life expectancy. They described this in terms such as “*their world crashing down, feeling guilty, and feeling sorry for him that he is losing out*”. Majority of the parents in this study expressed difficulty in obtaining a diagnosis for their child’s condition, and they described their journey as long and uncertain. When they received their child’s diagnosis of MPS, as a rare genetic life-limiting condition, they reacted with upset, devastation, anger and shock. It is not unusual for these parents to experience stress and anxiety about what the future will bring. All of the parents described the process of trying to accept their child’s diagnosis in terms of emotional struggle. One of the parents recalled her experience of receiving her child’s diagnosis of MPS:

*“We were devastated when we heard, we were shocked because we didn't think it was this condition at all, we knew nothing about this condition, never heard of it before.” (FA07, pp. 2) (F7, 1* ^*st*^ *Interview)*

*“You could dwell and dwell and think MPS, but you can't,” (F07), “Upset, confused, frustrated, I didn't know what was happening,” (F06).*

Families in this study disclosed their worry about watching their child failing to catch up with his/her peers, and feeling that their child was losing out on a normal life. They reported that their attention predominantly focused on their sick child resulting in their healthy child often being left out. One of the parents expressed concern about their healthy child, describing how hard it was to manage two lives at the same time:

*“Yes it is like having two lives and trying to balance them in the one house at the same time; it is just very, very hard. You have got one that needs constant attention, demands constant attention and you give it to them because they need it. But you also have another one who needs attention, but you can't give it to them because you think I will give it to you later, you'll be fine”. (FB04, pp. 20) (F4, 2* ^*nd*^ *Interview)*

Families spoke about the devastation of watching their child failing to grow up as a normal teenager, and the realisation that the gap would widen as he got older. They felt that their child was not leading a normal life and not doing the normal things that other children of his age were doing:

*“I know in years it will be getting harder as he is getting older, the things that he won't be able to do that his friends can do and stuff like that. You can see it now a bit, they want to be going from other places, and we say, ‘you can't be going.' Even going to a fun fair, a carnival, he can't go on the rides; he is not tall enough for this, and that and they are all going on. Things like that you can see changes in it that are the condition and how it affects him even friends going off places and stuff.”(FC03, pp. 8) (F3, 3* ^*rd*^ *Interview)*

The parents in this study described the dynamics of relationships as both a relational context and non-relational context. They reflected their experience with immediate family members, for example, husband, child, and close friends in a supportive care capacity and their relation with those in the healthcare system and services as a non-relational relationship status.

### Lived body

The existential theme of lived body reflects how the body is experienced, perceived, sensed and touched. According to Van Manen [31] (pp. 103), *“lived body refers to the phenomenological fact that we are always bodily in the world”.* The physiological impact of MPS is such that it has both visible and invisible clinical features. Two essential themes emerged from the lived body were: the stigma of a rare disease and MPS as encompassing multiple diseases.

#### The stigma of a rare disease

The clinical features of MPS can have a profound effect on the lived body, and that can lead to stigmatisation of children with MPS and indeed their parents. As a rare disease, the effects of MPS conditions can vary from one type to another. However, nearly all forms of MPS have physical characteristics that potentially cause visible stigma. Common features across types of MPS include a big head with depressed nasal bridge, thick bulging lips, enlarged tongue and coarse straggly hair – visible signs which contribute to the characteristic look of these patients. Children with MPS may also have concealed symptoms eg: learning disabilities and this can cause invisible stigma.

Parents in this study spoke about their fear of the visibility of MPS and how clinical features that were not obvious at birth gradually became prominent as they became older:

“*Yes diagnosed at 3½, yes it is weird enough because you go back thinking how come it wasn't picked up at the start? I suppose you never check for these things because he looks normal, he is born normal, normal weight. His features probably hadn’t come out by then …”. (FA03, pp. 13) (F3, 1* ^*st*^ *Interview)*

Another parent spoke about their realisation that some warning signs were visible from the beginning, but went unrecognised since their child was reaching all the developmental milestones:

*“Because we know what we know now we can see certain signs were there from [their child’s] birth like the hairy back, the protruding stomach … but they were just little things. Development wise he was hitting all the milestones, everything like that, so we didn't think anything was wrong. And then when we did get the diagnosis, yes, it is hard to accept there is something wrong with your child.” (FB02, pp. 3) (F2, 2* ^*nd*^ *Interview)*

Parents in this study felt that their child was not treated as ordinary by the general public, and the public’s reaction to them was considered as stereotypical and negative. Parents spoke about how individuals looked at their children’s peculiarities, and how they sensed that there was something wrong with their children.

As one of the parents stated:

*“People stare at children a lot, and they look at him funny. Kids can be ok; it is adults that are worse. ‘What is wrong with him, and what will happen to him, will he grow?' They are very nosey; they just want to know things but things like that annoy me, the way they talk. But [child] is great; he gets on with it…Things like that are a bit tough with him.” (FA03, pp. 3) (F3, 1* ^*st*^ *Interview)*

The need for parents to protect their children was clearly evident in this study. Parents felt that it was their responsibly to protect their children from the prying eyes of society. Parents narrated their experience of not treating their child any differently from others in society.

#### MPS as encompassing multiple diseases

Families described the experience of understanding different types of MPS such as MPS I-IV and VI, and they spoke about their awareness of the considerable variety of physical and mental characteristics presented by patients. They were able to identify which types of MPS were associated with normal intellectual functioning and which were not. The family of a child with MPS III compared their child with children who had other types of MPS:

*“And then people with MPS6, 4 and 1, they can join the conversation from early years up until the later years, adulthood, and join in the conversation and even some of them have a drink and all. Whereas we go from dealing with a very hyperactive child to progressing into a child who can't speak, no understanding and is 24–7 dependent. So you lose out in a big way. It is all MPS but totally different diseases.” (FA04, pp. 13) (F4, 1* ^*st*^ *Interview)*

Likewise, a family of children with attenuated MPS I spoke about their feeling for children with other more severe types of MPS:

*“And you see other parents, I mean the MPS disorder is horrendous, certain ones, Hurler's, Sanfilippo, Hunter's, I mean we have lost so many children. And they have a short life expectancy. We feel luck that we got the ...., which sounds terrible because life expectancy is normal once you are managed and maintained. … that it is not cancer, that is it not Sanfilippo, that it is not Hunter's, so that is probably a way of coping. We have probably taught ourselves to cope that way.” (FA01, pp. 16–17) (F 1, 1* ^*st*^ *Interview)*

There were two families in this study whose children had progressed to a stage where no further treatment options were available. This was particularly notable in the case of the young adult with MPS III (Sanfilipo):

*“I was fuming because kids with cancer, there is treatments available for some of these kids, whereas there is no treatment for our child. What is the difference? In fact, it is worse. I would rather [my child] be diagnosed with maybe a small tumour when my child was four. Our child could be in college now. Whereas MPS has just destroyed my child’s body …” (F B04, pp. 8) (F4, 2* ^*nd*^ *Interview)*

The existential theme of lived body reflects on the impact the disease has on the physical body. This theme was illustrated in parents’ reflections on the progressive nature of MPS in children and its impact on the family’s life. Language used to express this theme included descriptions of devastation, guilt, concern about the impact on their child’s ability to engage fully with life, and their fear of how the increasing evidence of visible clinical features of MPS reflected their child’s bodily experience of this condition.

### Lived space

Space was another important theme, which emerged in relation to the transitional process the families were experiencing. They spoke interchangeably about time and space, with spatiality considered as a ‘*no man’s land*,' where they felt they were oscillating between the past, present and future due to the uncertainty in their child’s life. Two main themes emerging in relation to space were: Unknown future, and hospital vs. home.

#### Unknown future

Families described their experience of living in a space in which the future is unknown from the time that they were told of the prognosis and outcome for their children. Parents in this study spoke interchangeably about time and space. They felt that living with an unknown was a horrendous experience because of the limited treatment option. They described this experience of space as being in no man’s land or being trapped. These families described their lived space as uncertain, ambiguous and stressful, knowing that their child is living with a condition with a limited treatment option.

Families described their experince of powerless and endless worry about their child’s prognosis and the progressive nature of the condition. One parent described the experience of being trapped eloquently:

*“It is a constant worry. You are constantly worrying about what they are going to say because you are afraid that you have missed something that they have seen, and you just feel like your life is trapped. You feel like you are in a box, and you just can't get out of it”. (FC06, pp. 9)(F6, 3* ^*rd*^ *Interview)*

Families described their experience of living in an unknown future from the point of which they were told of the prognosis and outcome for their children but also as a cyclical process, with the same fears and worries coming to the fore each time they engage with services.

*“The future is unknown because every time we go to see a doctor, like today I am going to see a neurological doctor with my son, you worry every time you go to see a doctor because you don't know what they are going to find or not find.” (FA01, pp. 3) (F1, 1* ^*st*^ *Interview)*

“No man’s land” is a term used by many families in this inquiry as a way of describing their experience of living with this condition. This space is described as a lonely and unending journey. Such feelings of uncertanity are prominent in this group of families who face the transition stage where they no longer belong to the previous status, yet have not completed the passage to the next [36, 37]. Parents described their life as oscillating between the past, present and future space of uncertainty in relation to their child’s life.

*“Fine if your blood pressure is high or low because you can cope with something that is within somebody's grasp, but that was way beyond both our grasps. It was a horrendous time, I have to say, horrendous because when we found out about it, there was no cure, there was no treatment, so we were in no man's land. They are now on treatment but back then there was no treatment. Looking back now there was trials that we were unaware of that were happening, but it meant nothing to us”. (FA01, pp. 6) (F1, 1* ^*st*^ *Interview)*

From the families’ perspective, they are living in a space of ambiguity, where they don’t know what is ahead and described feelings of being trapped in a box or square that they couldn’t get out of.

#### Hospital Vs. Home

The concept of space in hospital versus space at home was a notable factor in the lived experience of parents in this study. Unlike a parent of a child with a temporary illness, such as the flu, parents of children with MPS have to adapt to the fact that their illness is chronic and may deteriorate. These children and their families spend significant periods of time in hospital. All the children except one were initially treated with weekly Enzyme Replacement Therapy (ERT) in the national metabolic service. At the time of the study, five children were receiving weekly ERT at home. Three of the children with MPS I (Hurler syndrome) in the study had received Bone Marrow Transplant (BMT) outside Ireland following an initial period of ERT treatment. Parents of children receiving home treatment expressed satisfaction with moving from weekly hospital ERT to home-based treatment. The subthemes that emerged from the parent's own expressions are: hospitals bring you back to reality, you are in your home and do your own thing, and the healthcare system as a revolving door.

Families reported positive effects of home ERT when they compared their weekly hospital commitment to treatment for their children. They preferred to be in their home rather than a hospital, and they felt being in their particular home aided the development of routine and structure in their regular life. This parent expressed her experience of being at home for her son’s ERT:

*“You are in your home and do your own thing and get on with it if you need to. You are not waiting in the hospital all day long; you can be there getting the dinner or doing the household chores and stuff. And he is more relaxed at home because his friends can call and that, now they are on the summer holidays they can play away and stuff.”(FC03, pp. 4) (F3, 3* ^*rd*^ *Interview)*

The family of a child with MPS I who went for BMT outside Ireland expressed how they felt great to be coming back home after six months of treatment. They also spoke about the frustration of leaving family behind for six months to go outside Ireland to get treatment for their sick child.

*“But it was just great to be home. After being away nearly for six months it was different coming back then, but it is just great to come home.” (FB07, pp. 5) (F7, 2* ^*nd*^ *Interview)*

Families described hospital as a wide-open space that made them feel exposed to the reality of having a child with MPS. They felt that healthcare professionals’ knowledge on MPS might be limited considering the rare nature of the disease. They recommended having a dedicated space such as a centre of excellence of rare diseases, to facilitate education, training, and research and expand knowledge and practice in this area:

*“I think we do need our centres of excellence to be tapped into the centres of excellences of every other country so that we are teaching each other because this is all teaching … These are all the things we have to share because our society of these conditions is too small within certain countries. So definitely I think, and the world is built that you can do that, you can share the information with hospitals.” (FB 01, pp. 16) (F1, 2* ^*nd*^ *Interview)*

*“There has to be a centre of excellence, 100 % and I do believe, I mean with MPS and other conditions …” (FB01, pp. 15) (F1, 2* ^*nd*^ *Interview)*

Families spoke about their experience of the home as a space of comfort or contentment. When they spoke about their home they described feelings of security and protection. In this study, families disclosed their experince of having spent time outside the country away from home and they described feeling a sense of belonging and comfort when they returned to their home and were united with their family members. There was also a sense that MPS and its progressive nature changed families’ sense of space, as their priorities change. One of the families shared their experince of adapting their house and room to suit their child’s ongoing behavioural challenges and safety issues.

Families reported that they felt vulnerable due to that fact that collaboration and communication between healthcare service providers are often fragmented and unsatisfactory. They expressed disbelief and frustration towards healthcare systems and felt that these systems had little understanding of what challenges parents of children with MPS experience in their day-to-day life. They described the current healthcare system as being like a revolving door.

*“So you go through, it is like a revolving door, you go in, you go out, you go in, you go out, and you are only the number of the day, and the doctor and the patient can make it special. But to the system, I don't think it is a caring enough system.” (FB01, pp. 17) (F1, 2* ^*nd*^ *Interview)*

One of the parents described feelings of anger, frustration, and disbelief towards the health service, and she believed that system had no understanding of families day to day experience. There were numerous occasions she “*just felt like walking, just going away and never coming back”,* and she described the process by which decision were made by the healthcare system as taking too long.

*“So it is frustrating that this process is taking so long. I would love like if there was a miracle like I know he can't be put on this treatment willy-nilly, there is a process, but the process, from a parent's perspective, is so long, it is taking far too long to be sorted. There were times I just felt like walking, just going away and never coming back; it has just been so hard.” (FC02, pp. 5) (F2, 3* ^*rd*^ *Interview)*

Families reported numerous challenges and frustrations with the current health system. They reported that corordination and communication between healthcare services are fragmented. For example, families reported, “*trouble from one hospital getting an x-ray to another hospital*.” They also communicated concern over shortcomings in the current healthcare services, which intensified their apprehensions regarding their child’s future. Families in this study spoke interchangeably about time and space. The existential theme of lived space rather than mathematically measured space expressed how these families experienced different spaces in their day-to-day lives.

### Lived time

The existential theme of lived time describes how time is experienced in the parents’ day-to -day life caring for their child, adolescent and young adult with MPS. There was a reciprocity in how parents talked about time and space. They described being frightened as a result of the lack of cure or treatment for their child’s illness, and the feeling that the future is obscure to them and their children. They described periods of waiting as distinct from periods in which they are actively involved in engagements. The following themes emerged from lived time: the experience of waiting and a tough road ahead. Families spoke about their day-to-day lived time, their hope, expectations, and the uncertainty of their life to come.

#### Experience of waiting

Parents in this study reported the frustration of waiting for a long time for a diagnosis, to get a medical card for their child and to get basic needs like a wheelchair for a child with a physical disability. Early diagnosis and intervention in rare diseases can improve an individual’s life expectancy and QoL. It can also provide important information to guide further reproductive choices for the family. The subthemes emerging from parents expansions were noted as; you are left waiting, waiting and waiting, and it’s like watching a time bomb.

*“Yes, it is tough although we expected it, we have been told it would happen, but you are never ready to hear it …And it is just so frustrating, and it is sad to see everything being taken away from him, the work we put into doing what he does now and what he did before. And it has just been taken away from him bit by bit because the condition is eating away at him.” (FC06, pp. 4) (F6, 3* ^*rd*^ *Interview)*

Families experienced difficulties in accessing additional medical and non-medical services for their children. They spoke about spending significant amount of time navigating bureaucracy instead of caring for their sick children. One of the families discussed the issue of waiting to get a wheelchair for their child at the first interview in December 2013, and they were still waiting for the wheelchair at their 3^rd^ interview, a year later:

*“Yes, we are still waiting on our wheelchair. There was supposed to have been an assessment done in August, and he [husband] got sick again when he came back home he got sick again, and I had to ring them and cancel them, and they said they would send me out an appointment. It is only now I realise they haven't sent an appointment.” (FC04, pp. 32) (F4, 3* ^*rd*^ *Interview)*

Families described the emotion of watching their child’s progression as like watching a time bomb. A parent recounted her experience:

*“…Any parent who is given a diagnosis of their child having a life threatening condition, a terminal condition, of course, your world is going to come crashing down because you don't know what is ahead of you. And that is the thing; we know this condition is progressive, we don't know when [son] will start to deteriorate. It is just like watching a time bomb. Like every little thing, you are wondering is this the start of it or whatever…” (FB 02, pp. 4) (F2, 2* ^*nd*^ *Interview)*

They spoke about their collective experience of waiting: waiting for their child’s diagnosis, waiting to access expensive ERT treatment, and persistent waiting to get additional support mechanisms from the government agencies.

#### A tough road ahead

In this study, families expressed subjective time opposed to clock time. They spoke about their day-to-day lived time, their hopes and expectations. Their experience showed that lived time and lived space are mingled; they spoke about the uncertainty in their life and not knowing what to expect.

Families expressed their powerful emotions at the different stages of their child’s illness when they found out MPS is a progressive life-limiting condition with no curative treatment options available at that time. Families expressed their emotion of watching their child’s progression as like watching a time bomb. A parent recounted her experience:

*“…Any parent who is given a diagnosis of their child having a life threatening condition, a terminal condition, of course, your world is going to come crashing down because you don't know what is ahead of you. And that is the thing, we know this condition is progressive, we don't know when [son] will start to deteriorate. It is just like watching a time bomb. Like every little thing, you are wondering is this the start of it or whatever. And then the only thing that gives you hope is when he comes to his check-ups and you get great news you know he is ok.” (FB 02, pp. 4) (F2, 2* ^*nd*^ *Interview)*

Family of the young adultd with MPS III spoke of how their child had continued to deteriorate over the years, and this was clearly evident from their multiple interviews.

*“It changes over the years. The child we have now, even though our child is 20 is a completely different child that we had when our child was 3, when our child was 9. It changes completely and it is very hard to realise that you have to change with our child… Yes, the condition is probably less mentally draining now and more physically challenging”. (PA04, P-3) (FA04, pp. 3) (F4, 1* ^*st*^ *Interview)*

*“…So we probably feel a little more vulnerable now, and a little more shook up because the condition has very gradually changed from day one.” (FB04, pp. 3) (F4, 2* ^*nd*^ *Interview)*

Regardless of their worry about their children's future and their knowledge of the tough road ahead of them, the families were also living in a present time. They considered themselves frequent flyers who lived life day by day. The existential theme of time described how time is experienced in the parents’ day-to -day life caring for their child, adolescent and young adults with MPS. Van Manen argued [32], (PP.306), *“we experience the time of waiting differently from when we are actively involved in something”,* and he also highlighted that lived space and lived time are often interlinked. Parents talked about time and space in a reciprocal way and described finding their lived space frightening as a result of the lack of cure or treatment for their child’s illness and the fact that the future is obscure to them and their children [38].

### Lived things

The existential theme of materiality reflects how things and technologies are influenced in thier day to day life while caring for their child. Van Manen [32] argued that it would be difficult to overestimate the significance of things in our day-to-day life. Lived thing comprised a theme that was reflective of the impact material things had on the phenomenon of parents’ experience of living with and caring for MPS – i.e. things in their day-to-day life with MPS.

#### Things in their day-to-day life with MPS

Families described how material objects, for example, phone, the internet, hoist and wheelchair were devices that reinforced the reality of their living with a child who had complex needs, and how such objects could impact negatively or positively on their life.

One of the families indicated that they found a hoist as a great help when it came to caring for their young adult, who was physically challenging for the parents. They spoke about things in their day-to-day lives, such as nappies, wipes, and wheelchairs. They also pointed out that how important it was for them to have a wood-burning stove, because their child was very prone to hypothermia:

*“The hoists are great, but it still involves a bit of lifting and what have you. But that is where we are at the moment, she is just physically challenging more so, and it requires teamwork. Even going on a trip in the car, it is like packing to go on a holiday sometimes with nappies, wipes, feed, emergency stuff and then getting her into the wheelchair van, up the ramp, securing her. It is just … When you are going out for a day you make the most of it.” (FA04, pp. 4) (F4 1* ^*st*^ *Interview)*

It has been reported that searching for easily understandable rare disease-related information by individuals and their family members can be difficult, and when information is found it can be a frustrating and scary experience [25]. Families reported on the strengths as well as the risks of internet usage in searching for information on rare disorders. On numerous occasions, they felt the internet could be a wonderful tool, and they also spoke about the internet as a dangerous tool.

When a child is diagnosed with a rare genetic life-limiting illness, parents are regularly pushed into a whirlwind of feelings. Experiences around the time of the diagnosis were discussed earlier, but here we focus on the manner in which the diagnosis was disclosed or managed by the healthcare professionals over the telephone. This mode of communication can have a critical and substantive effect on parents’ psychological wellbeing. The majority of the families spoke about their dissatisfaction with the manner in which the diagnosis of MPS was disclosed over the phone. They felt it was horrendous, and predominately spoke about how the “thing” (i.e. the “phone”) affected them in their day-to-day life. One parent described this experience as follows:

*“Over the phone it was horrendous for the reason being I had no support around me, I was at home, the children were at school or crèche or wherever they were and the name Mucopolysaccharidoses was way beyond my comprehension of a word I had ever heard of before. It sounded big, and it sounded serious and it was just an awful way to be told. Automatically I said, ’ have to get my husband to ring you.' Because I couldn't take it in, even though I didn't know what it meant, I knew it didn't mean something good.” (FA01, pp. 6) (F1, 1* ^*st*^ *Interview)*

Likewise, another family expressed recollection of and their experience of hearing their child’s diagnosis of MPS:

*“He said he would let us know, but then he rang. It was bit weird when you think of it. Then we had to go for a meeting with him anyway but he had actually told us over the phone. It is probably not the right way to tell you something, is it? It would be better off going into his office and putting it all in front of you. But he would tell you again, as I said we went off then of course but by the time we got to see him we had all these things got, people had got stuff off the internet for us, booklets, downloading whatever all and printing off sheets. Maybe you are better off coming in and discuss what is wrong or whatever; we would have been better off that way. Because you are looking it up and it is not great.” (FB03, pp. 4) (F3, 2* ^*nd*^ *Interview)*

Families spoke about how the internet has become a helpful tool and their experience of searching on the internet and looking for information on their child’s illness following diagnosis. Some of the parents in this inquiry use Facebook as a mode of communication and update, and they found it very beneficial for networking and supporting other families with a similar rare condition like MPS.

## Discussion

The purpose of pursuing this inquiry was to gain a deeper understanding of what it is like to be a parent of a child, adolescent or young adult with MPS. The main themes emerged from this study were described as living with MPS, living with a genetic rare disease, the stigma of a rare condition, MPS as encompassing multiple diseases, Unknown future, hospital vs. home, experience of waiting, a tough road ahead, and things in their day-to-day life with MPS. The majority of families started their lived experience from the time they received their child’s diagnosis and this diagnosis then impacted their life as a whole. They spoke about emotional reactions and coping strategies associated with both diagnosis and the ongoing challenges of living with a progressive condition that has no cure. They spoke of their child’s QoL, their healthy children’s wellbeing, and for some, the impact on their own physical and psychological wellbeing. They also reflected on issues of stigmatization and isolation in their experience of living with a child with a rare disorder. Even though the impact of child’s rare life-limiting illness on parents has been explored in a number of studies [13, 14, 18, 39–41] this study is the first of its nature that has explored the experience of Irish parents living with and caring for a child, adolescent and young adult across the full spectrum of MPS diagnoses.

This study’s findings reflect the wider literature looking at the impact of other types of life-limiting illness, which have also indicated that caring for someone with MPS has a wide impact across all dimensions of the family’s life [13, 14, 39–41]. These findings are again consistent with the literature on parents of children with other life-limiting conditions [13, 14, 39–41] and life-threatening conditions [42, 43]. Equally consistent with the literature was the picture that emerged of parents adapting and fitting in around their child’s increasing needs, as their illness progressed [13, 14, 44]. Families described their evolving role of a parent to a main care provider, with mothers acting as the principal care providers among those interviewed. Only one father was interviewed, although the invitation to participate was open to both parents, either together or separately. Fathers clearly play an important role in these children's lives, and take on roles of protecting and providing care for their family. However, the study findings are consistent with literature which shows that women continue to primarily take on the role of caregiver when a child has a life-limiting illness [45, 46]. The notable concern on the part of parents for their healthy children's welfare is expressed in this study and is mirrored in other literature [47–49]. Studies have consistently shown that having a rare disorder gives rise to family problems, strain on marital relationships, feelings of isolation and additional financial pressures, as well as having a negative impact on healthy siblings [12–14, 20, 50].

This study also highlights the inadequacy of communication skills among healthcare professionals, especially during the initial diagnostic disclosure. The study findings are consistent with other published studies on challenges in the way in which terminal illness diagnoses are disclosed to families [20, 51, 52]. The majority of the families in this study did not receive their child’s diagnosis of a rare life-limiting condition face to face in the clinic and were dissatisfied with how healthcare professionals disclosed their child’s diagnosis, particularly in instances where diagnosis was given over the phone. Of note, the majority of the families were not referred to, or attending, the national centre that provides specialist care for MPS at the time of diagnostic disclosure. There is the need for clear national clinical pathway or guideline to manage the assessment of diagnosis and breaking off bad news. This study also reported that the healthcare professionals’ lack of specialist knowledge of the rare condition raised challenges with diagnosis and appropriate specialist referral. There is a need to develop a competency framework for rare diseases to avoid any unnecessary delay in referral, diagnosis, and disease management or treatment.

This study highlighted the need for greater and more diverse initiatives that could serve as indicators for the future understanding and development of policy and practice related to paediatric rare life-limiting conditions. Even though parents reported some positive aspects of current services, e.g. their relationships with primary healthcare professionals and the quality of services they currently receive, the overall findings of the study highlight difficulties in accessing services. Parents described inconsistent and inequitable services often characterized by bureaucracy and delay. Despite findings from across studies [21, 39, 44, 51] there is a consistent shortfall in social, emotional and respite support for families of children with MPS. The study findings highlighting the need for more responsive physiotherapy, occupational therapy, psychology and social support services at the regional centres. The study findings reflect other studies which suggest that family needs are subject to change over a period [13, 14, 21, 53, 39, 44, 51]. Study findings highlight the importance of frequent and planned re-assessment of the social, emotional, and economic challenges experienced by children and their families [54]. The findings also point to unmet mental healthcare needs among parents. If these unmet needs are not addressed, there is a risk that these families will experience negative consequences additional to the already high burden and cost of their child’s condition. There is also need to set out a process framework on six monthly to annual review to assess and advise families needs and psychosocial support services in the care of children with rare progressive diseases.The findings of the study recommend having a designated key worker role in the care and management of children with MPS and related diseases. This position will help to enhance parents trust in the health system and overall coordination to minimise waste of time and resources.

Families reported that their experience of partcipating in the research interview was equal to a counselling session or as a therapeutic relief from their stress, but expressed a sense of limbo when the research interviews were completed. However, the purpose of the research interview was not intentionally offering any form of therapy, despite the participant report. This study suggests that researchers should be aware of the qualitative therapeutic interview process and its possible benefits for the participants’ emotional wellbeing throughout the research process, that is also mirrored in other literature [55, 56].

### Study limitations

There are some advantages as well as disadvantages to using phenomenology as a research methodology. The subjectivity of the data could lead to difficulties in establishing what may be considered a more generalised approach to reliability and validity. Further, the small sample size and uniqueness of lived experience can be challenging in making an inference for a wider cohort or group. There were consequent organisational changes following recommendation made in the national plan for rare diseases [23], and the recent establishment of an adult metabolic centre may have created some uncertainties among these families. Finally, as already acknowledged, this study provided an interpretation of the lived experience of parents of children, adolescents or young adults with MPS at particular moments in time, and not at the point of diagnosis. It also did not follow parents through the end stage of their child’s condition. A study that adopted a strategy of longitudinal engagement over a longer period, including both diagnosis and death, while posing challenges in terms of feasibility would be optimal for a complete understanding of the phenomenon.

### Future research

The study suggested that there are many aspects of the care of children, adolescents or young adults with MPS that require further research attention. This study included serial interviews spanning a time period of one to two years, and as such attempted to capture the progressive nature of this illness and its impact on children and their families. However, the findings point to the need for future research into the experience of living with MPS from the children’s or individuals’ perspective, specifically to investigate various aspects of this chronic debilitating illness. These aspects might include how the patients perceive themselves (self-image), how the child or adult with MPS copes with issues such as stigma and engagement with services, and what they feel about ERT or BMT. To date, the healthcare professionals caring for the children with MPS have not been afforded the opportunity to describe their experience of caring. To address the lack of formal research into the healthcare professionals’ experience of caring for the children with MPS, this study recommends further research into health professionals’ perspective to complement the findings of the current study. Further research is also recommended to explore the level of support each separate family member requires, including healthy siblings and grandparents, as well as investigating the needs of families in relation to the long-term care of their child through adolescence to adulthood and the specific services required at different points of the disease cycle.

## Conclusion

This research study is the first of its kind to act as an initial enquiry and to generate knowledge through researching the lived experience of Irish parents’ of children, adolescents and young adults with MPS. This study provided a voice to the Irish parents of children with MPS, and in doing so will make their lives more understandable to the wider audience. It brings to light the uncertainty, sorrows, and everyday challenges faced by these families, and hopefully will improve the care and support for them through the many months and years of their child’s illness.

## Abbreviations

BMT: Bone marrow transplant
ERT: Enzyme replacement therapy
GAG’s: Glycosaminoglycan’s
HSCT: Haematopoietic stem cell transplantation
IMDs: Inherited metabolic disorders
MPS: Mucopolysaccharidosis
NCIMD: National Centre for Inherited Metabolic Disorders
QoL: Quality of life
UCBT: Umbilical cord blood Transplantation
